# Integrating the Essentials Core Competencies Related to Health Literacy Into Undergraduate Curriculum: Tapping Traditional and Emerging Education Strategies

**DOI:** 10.1111/phn.70041

**Published:** 2025-11-24

**Authors:** Karen L. Valcheff, Deidra S. Pennington, Sarah K. Keaton, Kimberly Ferren Carter

**Affiliations:** ^1^ College of Nursing Radford University Radford Virginia USA

**Keywords:** artificial intelligence and public health and artificial intelligence and reading level, artificial intelligence, *Essentials*, health literacy, nursing students, tools artificial intelligence and foreign language

## Abstract

The COVID‐19 pandemic exposed weaknesses in healthcare systems regarding health literacy. Although involving individuals, families, and communities in healthcare decision‐making improves their outcomes, their ability to interpret information depends on attention to health literacy and readability of materials. Nursing educators have an essential role in teaching students how to implement literacy‐appropriate health education strategies. The American Association of Colleges of Nursing (AACN) *Essentials: Core Competencies for Professional Nursing Education* includes health literacy content in the domains, competencies, and sub‐competencies. There is a dearth of literature that guides faculty to apply the *Essentials* related to health literacy. Grounded in the health literacy components of the *Core Competencies*, this paper presents traditional, artificial intelligence (AI) enhanced, and interprofessional education (IPE) teaching strategies and methods for evaluation to develop health literacy competency. This paper will be useful for faculty who desire to integrate the core competencies with innovative approaches to teach health literacy within the undergraduate nursing curricula, to address health literacy disparities, and promote equitable, positive individual outcomes.

## 1 Introduction

Health literacy education is the cornerstone for positive individual outcomes. Health literacy differs from the idea of being literate, which is the ability to read and write. There are many aspects to health literacy, including personal, organizational, digital health, and numeracy (NLM 2024). According to the National Library of Medicine (NLM 2024), “Personal health literacy is the degree to which individuals have the ability to find, understand, and use information and services to inform health‐related decisions and actions for themselves and others” (para. 2). Organizational literacy involves institutional support provided to individuals promote access to care and decision‐making, while digital health literacy involves the use of a variety of electronic tools and information (NLM 2024). Numeracy, or quantitative literacy, involves applying analytical calculations and reasoning to improve personal health outcomes (NLM 2024). Most individual situations in nursing practice involve the interplay of personal, organizational, digital, and numeracy literacy. For example, individuals with diabetes must have access to (organizational) and be able to use a glucometer (digital) to interpret their blood glucose level and understand how to adjust their medication based on the interpreted blood glucose value (numeracy). Incorrect interpretation can lead to incorrect dosing of their insulin, causing potentially devastating health outcomes (personal).

To have optimum outcomes, it is necessary for the individual to understand and utilize the information provided by the health team. Those with low health literacy seek medical care later and present with more complex health issues (Aljassim and Ostini 2020). Poor health literacy correlates to the misuse of medications, missing medical appointments, and not fully understanding the plan of care (Kim et al. 2022). Undergraduate nursing education plays a pivotal role in preparing the next generation of nurses to provide individual care that is sensitive to health literacy. Grounded in the health literacy components of the American Association of Colleges of Nursing (AACN 2021) *Essentials: Core Competencies for Professional Nursing Education* (referred to *Essential*s in this paper) domains, competencies, and sub‐competencies, this paper presents traditional, artificial intelligence (AI) enhanced, and interprofessional education (IPE) teaching strategies and methods for evaluation to develop health literacy competency. There is a need for literature to guide faculty in both the application of the *Essentials* and in specific strategies to address health literacy competencies in undergraduate nursing education. This article can serve as a template for nursing educators to use to incorporate the *Essentials* into curricula. A literature search of the databases CINAHL, PubMed Central, and Google Scholar was performed using the keywords: AI, health literacy, nursing students, *Essentials*, tools, AI and foreign language, AI and public health, and AI and reading level. The article review was limited to those available in the English language, from peer‐reviewed journals, and published within 5 years, except for foundational articles. The literature review identified key strategies for nursing educators to incorporate into clinical and didactic settings to aid the development of health literacy competencies within the framework of the *Essentials*. The incorporation of AI strategies is a timely consideration as leading nursing organizations are recommending the incorporation of AI into nursing curriculum (National League for Nursing [NLN] 2025). Additionally, the identified models and toolkits will provide educators with the structure to develop strategies for application in clinical and classroom.

## 2 Background

The World Health Organization (2023, 2024) has deemed health literacy a global health issue. In the United States, approximately 88% of adults lack sufficient health literacy, reflected by 55% of adults with intermediate proficiency in understanding basic health information, 22% with basic proficiency, and 12% not meeting a basic proficiency level (Lopez et al. 2022). Low health literacy is associated with more hospitalizations and greater use of emergency care for nonemergent issues (Shahid et al. 2022). Ziapour et al. (2022) found several factors that can impact health literacy, including “gender, education, occupation, income, and place of residence” (p. 1). Regardless of literacy level, stress from acute illness or chronic disease can cause increased anxiety and confusion, resulting in misinterpretation of information or decreased health literacy (Dehghani 2024).

Poor health literacy is a driver of inflated healthcare costs in the United States (Shahid et al. 2022). The US national health expenditure increased 7.5% (4.9 trillion dollars) in 2023 (Centers for Medicare and Medicaid Services [CMS] 2024). In addition, CMS identified an increase of 1029.8 billion dollars in Medicare spending and 871.7 billion dollars in Medicaid spending in 2023. When compared to other wealthy countries with similar economies, the United States spends almost twice as much on healthcare costs per individual (Wager et al. 2025). Blumenthal et al. (2024) evaluated the healthcare systems of 10 countries with economic stability, like the United States, for performance in “access to care, care process, administrative efficiency, equity, and health outcomes” (para. 3). Compared to the other nine countries, the United States ranked last overall despite increased healthcare spending (Blumenthal et al. 2024). Low health literacy levels contribute to these increased healthcare costs and negative individual outcomes (Lopez et al. 2022). A key driver to poor outcomes is the individual's lack of understanding of the healthcare system and the quality of education provided by the healthcare team. By teaching undergraduate nursing students the *Essentials* core competencies related to health literacy, educators can prepare students to make a meaningful impact on healthcare outcomes.

## 3 Health Literacy‐Sensitive Curriculum Plan

*The Essentials* (AACN 2021) provides the framework to integrate innovative teaching and evaluation strategies for health literacy into baccalaureate nursing curricula (Appendix). This framework supports health literacy competency introduction, reinforcement, and mastery through repeated exposure; thereby empowering future nurses to communicate better with individuals, recognize diverse health needs, and promote informed decision‐making in a variety of healthcare settings. Four domains in the AACN *Essentials* (2021) have health literacy‐related components (Domain 2, 3, 4, and 8). Domain 2 focuses on person–centered care and the collaborative approach to individual wellness. Domain 3 addresses population health and the need for interprofessional partnerships to improve health outcomes for diverse populations. Domain 4 reflects scholarship for the nursing discipline and explores the elevation of care through the discovery of effective evidence‐based practices. Domain 8 concentrates on informatics and healthcare technologies and the use of data‐driven decisions to improve individual care delivery.

The role of faculty in health literacy integration is to create objectives, innovative teaching strategies, and evaluation methods. The Appendix can be used as a resource for curricular threading of health literacy. For example, in competency 2.2, sub‐competency 2.2e, the importance of delivering appropriate materials tailored to the target audience would be introduced to promote collaborative and person‐centered care. As an in‐class activity, students assess reading levels of brochures and education materials from local agencies using the *Simple Measure of Gobbledygook (SMOG) Index tool* (Harvard T.H. Chan School of Public Health 2025b). Use of the SMOG Index assessment tool provides an opportunity for nursing students to evaluate educational materials and determine if the materials meet the 4th to 6th grade levels as recommended by the Agency for Healthcare Research and Quality's (AHRQ) Patient Safety Network (Bakerjian 2023). In addition, the use of role‐play can be used to demonstrate difficulties faced by individuals with visual and auditory impairment. Using the *PEARLS Healthcare Debriefing Tool* (Bajaj et al. 2018), faculty can lead student discussions on the implications of distributing these materials to various populations and how to improve the delivery of health education materials. Students can then apply the concepts introduced through these activities with individuals in the clinical setting.

The COVID‐19 pandemic brought to light significant gaps within our healthcare system, including limited digital health literacy and the need for interprofessional teams to improve delivery and understanding of healthcare information (Aker et al. 2022; Ogrodnick and Feinberg 2023). Collaboration between healthcare providers, community partners, and individuals to develop interventions aimed at improving health literacy can improve outcomes and decrease healthcare costs (Hearn et al. 2023). Collaboration among the interprofessional team can establish shared healthcare goals for individuals, communities, or populations (Ruebling et al. 2023). The Appendix in *Essential 6.4d* highlights opportunities for teaching with interprofessional student groups. This could be offered in both clinical and didactic settings. Furthermore, the opportunity for nursing students to collaborate with students from various disciplines enhances their ability to adopt an interprofessional approach for future practice (El‐Awaisi et al. 2025). Engaging in community health visits with additional student disciplines allows students to view individuals through a holistic lens, improving health outcomes, and meeting their individual needs (David et al. 2024).

## 4 Resources for Educators

### 4.1 Models and Toolkits

#### 4.1.1 Health Literate Care Model

There are many models and toolkits that are valuable resources for educators. The *Health Literate Care Model* outlines strategies that can be employed across organizations and with the collaboration of community partners to improve individual health literacy outcomes (Office of Disease Prevention and Health Promotion [ODPHP] 2021). This involves approaching all individuals as if they do not understand basic health information (ODPHP 2021). Through a systems approach, the *Health Literate Care Model* outlines the necessity of individual engagement with the healthcare team and the benefit that community partners play in providing support to improve individual health (ODPHP 2021). The ODPHP (2024) provides resources for written, digital, and web‐based content and tips on implementing the *Health Literate Care Model* (ODPHP 2021).

#### 4.1.2 Health Literacy Universal Precautions Toolkit

Brevidelli et al. (2024) cite the benefit of using the AHRQ *Health Literacy Universal Precautions Toolkit* to aid in improving the delivery of healthcare information while making it accessible for all. While this toolkit is geared toward use in primary care practices, the principles used can be applied in other settings, such as educating students on how to develop and provide health information while considering the individual's health literacy. In a study by Bidlespacher and Mulkey (2024), a 45% decrease in rehabilitation readmissions occurred after implementing the AHRQ *Health Literacy Universal Precautions Toolkit*.

The first action in the AHRQ toolkit is to start the path to improvement, which includes forming an interprofessional team, developing a plan of action, and raising awareness (Brach 2024). Interprofessional collaboration can be reinforced in the clinical setting as well as the classroom. Interprofessional team home visits will allow students to visualize the positive impact of collaborative care on health literacy (Brevidelli et al. 2024). In the classroom, teams of students evaluate health information and discuss a plan of action on how they would address any concerns that arise. The teams present their findings and recommendations about how to integrate health literacy effectively when providing individual education.

#### 4.1.3 Material Assessment Tools

Tools frequently employed to evaluate educational material reading levels are AHRQ's (2020) Patient Education Materials Assessment Tool for Printable Materials (PEMAT‐P), the Flesch‐Reading Ease (Kincaid et al. 1975), the Fry Graph Formula Tool (Fry 1968), and the SMOG‐Index (McLaughlin 1969). These tools have been widely utilized for many years and remain popular on a global scale. The PEMAT‐P tool was created to evaluate the understandability of written material, while the PEMAT‐A/V evaluates audiovisual sources (Shoemaker et al. 2014b). Although an easy tool to use, the PEMAT has “strong internal consistency, reliability, and evidence of construct validity” (Shoemaker et al. 2014a, 1) and demonstrates a high degree of interrater reliability (Vishnevetsky et al. 2018).

The Flesch‐Reading Ease is used on a large scale among health professionals. The tool was originally created by Flesch in 1948, then adapted for use by the United States Navy in 1975 as the Flesch–Kincaid Reading Grade Level (Flesch 1948; Kincaid et al. 1975). Both versions are valid tools to test the reading levels of printed materials from fifth grade through higher education (Jindal and MacDermid 2017). Within Microsoft Word, users have the ability to activate the Flesch‐Reading Ease to evaluate documents for readability (Microsoft 2025).

The Fry Graph Formula Tool (Fry 1977) was created with the intent to maintain a user‐friendly approach while offering a broader scope of application on materials from first grade through higher education. In addition to healthcare, the validated Fry Graph Formula Tool has proven beneficial to other professions, for example, the evaluation of secondary education textbooks (Sarimanah et al. 2021). Since its creation in 1969, the SMOG tool has experienced widespread popularity. Elliot et al. (2025) recommend the use of SMOG as the preferred validated tool for healthcare literature because of its accuracy and ease of use. Ferguson et al. (2021) cite the ability of the SMOG tool to measure a higher level of comprehension as another advantage. Additionally, the researchers relay their choice of using the SMOG tool based on guidance from the Cochrane Collection (Ferguson et al. 2021; Cochrane Collaboration 2013). Rooney et al. (2021) justified the use of Flesch–Kincaid, Fry, and SMOG due to their well‐documented validation and the consistency of the results amongst tools.

#### 4.1.4 Additional Resources

The Centers for Disease Control and Prevention (CDC) created *The CDC Clear Communication Index* Tool (CDC 2025) to help guide the development of public health materials and provide evaluation for clarity and readability. The National Institutes of Health (NIH 2021) *Clear & Simple* guidelines include a five‐step approach to developing materials for individuals. These steps are: “Define the Target Audience, Conduct Target Audience Research, Develop a Concept of the Product, Develop Content and Visual Design Features, and Pretest and Revise Draft Materials” (NIH 2021). The website of Harvard's Center for Health Communication offers many useful aids that help improve numbers in communications, tips for creating presentations, and methods for evaluating the readability of materials (Harvard T.H. Chan School of Public Health 2025a). The American Academy of Ambulatory Care Nursing (AAACN 2023), provides links to websites, books for reference, online health literacy videos, and information on readability formulas to assist in the education of healthcare professionals.

The trend of individuals seeking medical advice on the internet has grown significantly in recent years (Van Bulck and Moons 2024). The term Dr. Google, gained popularity around 2010 and continues to be a main source of health information (Van Bulck and Moons 2024). Dr. Google integrates health advice from non‐medical sources found on the internet with the medical advice provided from credible websites (Millenson 2025). As a result, the information obtained may be unreliable. The Appendix in *Essential 8.3d* incorporates a teaching strategy to help students compare and contrast internet‐based information to evidence‐based recommendations.

### 4.2 Artificial Intelligence

The COVID‐19 pandemic has transformed how we approach healthcare through the use of Digital Health Literacy (DHL) and AI within healthcare and education (Boshnjaku et al. 2025). AI is the process of implementing technology that replicates “intelligent behavior” and “critical thinking” to provide human‐like problem‐solving responses (Amisha et al. 2019, 2328). Labrague et al. (2025) identified three AI strategies to enhance student learning: “AI‐driven simulation‐based learning, AI‐augmented instruction, and AI‐generated content and tools” (p. 3). AI‐driven simulation‐based learning aims to improve student knowledge using software‐based AI virtual simulations (Benfatah et al. 2024) and smart manikins (White et al. 2024). AI‐augmented instruction allows students the freedom to use AI software to expand assignments (Labrague et al. 2025). Using AI‐generated content and tools, faculty create learning activities including case studies (Higashitsuji et al. 2025), videos (Reed and Dotson 2024), and imagery (Reed et al. 2023) to aid student learning.

In academia, faculty play a key role in identifying the specific learning needs of students and helping to develop a unique plan that aids in achieving learning outcomes (Bosun‐Arije et al. 2024). AI has the potential to enhance curriculum development and prepare students for a future in healthcare (Simms 2025). The complexities of applying AI in a nursing curriculum and its use can be challenging for both the student and faculty (Porter and Foronda 2024). Incorporating AI information at all levels of a nursing curriculum allows faculty to build upon and teach AI concepts effectively and reduce the intimidation of AI use. AI does not replace the need for faculty involvement in the learning process (Bosun‐Arije et al. 2024; Tseng et al. 2025). In fact, faculty should remain vigilant to ensure the integrity of information being produced is accurate.

It is important for educators to implement AI strategies that allow students to augment their intellect but not rely fully on AI‐produced text for the entirety of the assignment (Bosun‐Arije et al. 2024). Public health nurses have the opportunity to integrate AI into their practice to improve health literacy. Panteli et al. (2025) recommend the use of AI in writing public health literature. AI language generators can effectively identify different reading levels and translate text into many languages (Panteli et al. 2025). By incorporating a student assignment that compares and contrasts the differences in reading levels of individual education materials facilitated by traditional tools, to the materials created with AI‐generated text, students will utilize critical thinking to analyze the results (Appendix 2.8d). Public health nurses often need the use of interpretive services for translating health information to individuals who speak a variety of different languages. An additional assignment that utilizes AI foreign language generators can help the student practice communication with individuals about their health information. Students can brainstorm how to verify the quality of messages produced, including examples such as possible family members, bilingual students and faculty, healthcare interpreters, and online interpretive services (Appendix 2.2e). After completing their projects, students would then collaborate with peers to review the materials and compare results.

#### 4.2.1 Ethical Considerations of Artificial Intelligence

There are ethical considerations that must be considered when integrating AI into an undergraduate nursing program (Bosun‐Arije et al. 2024; Porter and Foronda 2024; Simms 2025; Sumengen et al. 2025; Tseng et al. 2025). Faculty must take proactive steps to develop policies that reflect the ethical use of AI. Similar to an honor code disclosure, an AI disclosure can outline the acceptable and unacceptable uses of AI (Bosun‐Arije et al. 2024). An addendum to the honor code policy should contain a disclosure that students must sign each semester. It is the responsibility of the faculty to remain current in AI practices and ensure that students are utilizing the technology ethically.

Students have easy access to an increasing number of AI products on the internet, such as ChatGPT (Chang et al. 2024), SafeBot (Rodriquez‐Arrastia et al. 2022), GhatGAi (Kong et al. 2024), and ERNIE Bot (Kong et al. 2024). As AI quickly emerges and evolves, there is much work to be done to understand how to incorporate AI into student learning and the measurement of relevant outcomes. Additionally, it is important to monitor the impact AI has on nursing students’ NCLEX exam scores in the future.

The algorithms used in traditional AI follow a set of rules that help guide decision‐making (Simms 2025). A more recent evolution of AI involves generative software with the capability to produce more complex content (Simms 2025). It is within this complex content that new ethical dilemmas arise (Porter and Foronda 2024). Many of the algorithms introduce bias by overlooking individualized plans of care within marginalized and diverse populations (Porter and Foronda 2024). The implementation of AI algorithms should not diminish a nurse's capacity to make independent clinical decisions about individual care (ANA 2022). The key principles of “beneficence, nonmaleficence, autonomy, and justice” are essential to consider when developing student learning activities and reviewing AI‐generated content (Bosun‐Arije et al. 2024, 2). This is an especially important consideration for faculty teaching nursing students to critically consider the appropriateness of AI tools within the context of population health literacy needs.

## 5 Conclusion

As the healthcare system continues to evolve, innovative approaches for screening and the implementation of health education materials to improve health literacy are imperative. Health literacy is a Healthy People 2030 objective (ODPHP n.d.), further underscoring the importance of integration of health literacy concepts throughout professional nursing education curricula. To improve individual outcomes, there is a need for increased collaboration between the healthcare professional and the individual (Wilandika et al. 2023). For this to be accomplished, individuals must be able to understand and interpret their healthcare information. This paper offers innovative approaches to teaching and evaluating undergraduate students to achieve health literacy core competencies. Incorporation of the health literacy‐related components of the AACN *Essentials: Core Competencies for Professional Nursing Education*, use of creative learning experiences, such as the suggestions in this paper, will strengthen the nursing student's ability to provide sensitive and competent care. These suggestions are not intended to be exhaustive, but rather illustrative of how to approach the curricular threading, sparking creative ideas for each academic environment.

AI is rapidly transforming practice and education. With the many free resources available on the internet, healthcare providers increasingly refer individuals with lower e‐health literacy to open AI sources to find health information (Ayo‐Ajibola et al. 2024). Nurses should take the extra time needed to discuss the information found in AI sources and dispel any misleading notions the individual discovered. Individuals who use Dr. Google may need similar attention from the nurse. Guidelines from professional nursing organizations such as the NLN and ANA provide excellent direction for the use of AI in healthcare (ANA 2022; NLN 2025).

Nursing faculty should monitor how evolving AI information is vetted throughout undergraduate nursing programs and how students are utilizing that information to communicate with individuals. It is important to investigate how faculty implement AI and evaluate the associated student outcomes. Detailed policies for student learning in didactic and clinical settings need to include specific AI usage. Continuing education for faculty is necessary to ensure that current AI technologies integrated into the classroom reflect best practice. Collaboration with information technology specialists is important to receive the most current information about new AI systems. Regular review of accreditation guidelines is necessary to identify updates regarding AI use.

The role of the nursing faculty is to ensure the responsible use of AI and other tools to achieve health literacy objectives. Finding the balance between maximizing the potential of the technologies and maintaining ethical standards will require collaboration between policymakers, educators, practitioners, and students to create effective and transformational learning experiences (George and Wooden 2023). Referencing expert AI policies from the WHO (2023) and the US Agency for Research and Quality (Brach and Borsky 2020) will help guide safe practices for healthcare providers. Nursing faculty should keep abreast of new developments in AI policy for higher education. While policy‐making organizations throughout the world are grappling with AI policy, many of these are generic and without relevance to education, leaving universities to create rules and standards to meet their unique needs (Fu and Weng 2024). It is incumbent upon nursing faculty to ensure that these policies provide effective guidance and protection for faculty, students, and ultimately individuals.

Nursing educators are responsible for creating innovative educational activities within undergraduate nursing programs to address the students' knowledge gaps related to health literacy and technology. It is imperative to educate nursing students regarding the impact of health literacy throughout their individual populations, barriers associated with poor health literacy, and how to improve the efficacy of individual education relating to health literacy. Despite an abundance of research identifying the crucial issue of health literacy, a dearth of information exists regarding solutions to the problem. Appropriately threaded and developed content related to health literacy at the undergraduate level can prepare the next generation of nurses to address healthcare knowledge gaps and promote improved population health.

## Conflicts of Interest

The authors declare no conflicts of interest.

## Supporting information




**Appendix**: Educational Strategies to Meet Health Literacy Core Competencies.

## Data Availability

Data sharing is not applicable to this article as no datasets were generated or analyzed during the current study.
