# Supplementary material for: Integrating the Essentials Core Competencies Related to Health Literacy Into Undergraduate Curriculum: Tapping Traditional and Emerging Education Strategies
Source: Public Health Nurs. 2025 Nov 24;43(2):402–9. doi: 10.1111/phn.70041 (PMC12968492; doi:10.1111/phn.70041)
Supplement: Supplementary file 1 — Appendix: Educational Strategies to Meet Health Literacy Core Competencies. [file PHN-43-402-s001.docx]

**Appendix**

**Educational Strategies to Meet Health Literacy Core Competencies**

| **Domain** | **Competency/**  **Sub Competency** | **Objectives** | **Teaching Strategies** | **Evaluation** |
| --- | --- | --- | --- | --- |
| **Domain 2:**  **Person**  **Centered Care** | **2.2 Communicate**  **Effectively with**  **Individuals** |  |  |  |
|  | *2.2e*  *Use evidence-based* *teaching materials, considering health literacy, vision, hearing, and*  *cultural sensitivity* | Students will demonstrate evidence-based teaching strategies  Students will  develop culturally sensitive individual education materials | Simulation: Vision and hearing impairment (eg. glasses with Vaseline, earplugs, or background noise) while providing individual education  Simulation: Bilingual students act as individual and English-speaking students function as nurses delivering individual education  Simulation: Teach-back method for medication administration education  Interactive Class Activity: Use online foreign language generators to produce individual education reading materials in various languages (e.g. of online AI foreign language generators: Musely, Chat GPT,  Writify.AI, Blueue, and Cograder)  (Students will brainstorm how to verify the quality of messages produced  (eg. possible family members, bilingual students and faculty, health care interpreters and online interpreter services). | Faculty led debriefing with interactive discussion using the PEARLS Healthcare Debriefing Tool (Bajaj et al., 2018)  Student self-assessment/reflection  Student and faculty discussion about results |
|  | **2.8**  **Promote Selfcare Management** |  |  |  |
|  | *2.8b*  *Employ individualized educational strategies based on learning theories, methodologies, and health literacy* | Utilize the Health Literate Care Model in clinical interactions | Simulation: Education using Health Literate Care Model (Office of Disease Prevention and Health Promotion [ODPHP], 2021) | Faculty led debriefing with interactive discussion using the PEARLS Healthcare Debriefing Tool (Bajaj et al., 2018) |
|  | *2.8d*  *Demonstrate the appropriate use of health information literacy assessments and*  *improvement*  *strategies* | Student will appropriate-ly screen  individual materials using health literacy tools used in healthcare to determine reading level  Student will compare and contrast reading level results of identified health literacy tools to the reading level produced by an AI text level generator | Interactive Class Activity: Practice analyzing educational health pamphlets using AHRQ *Health Literacy Universal Precautions Toolkit* (Brach, 2024), SMOG tool (Harvard T.H. Chan School of Public Health, 2025b), Flesch-Kincaid Grade Level (Kincaid et al., 1975), Fry Graph Formula Tool (Fry,1968) and PEMAT (Agency for Healthcare Research and Quality, 2020).  Interactive Class Activity:  Practice analyzing reading levels on educational health pamphlets using online AI text level  Generators (e.g. online AI text generators: Musely, Chat GPT,  Writify.AI, Blueue, Word Document Readability Function (Microsoft, 2025),  and Cograder).  Interactive Class Activity: Small group discussion focused on the findings of the literacy tools, contrasting manual to AI generation | Student presentation to peers with peer and faculty feedback  Student presentation to peers with peer and faculty feedback  Student presentation to peers with peer and faculty feedback |
|  | *2.8e*  *Identify personal, system, and community resources available to support self-care management* | Students will identify roles and resources for client self-care manage-ment | Interactive Class Activity: Students revise health materials incorporating reading level assessment  Guest Lecture: Community Agency Presentation to students (e.g. health department, services for older adults)  Interactive Class Activity: Students work in groups to create a list of local, state, and national resources for clients to support self-care utilizing infographics and technology incorporating the knowledge learned through health literacy material assessment | Student self-assessment/reflection  Pre/Post lecture quiz to assess learning  Faculty led debriefing with interactive discussion |
| **Domain 3:**  **Population**  **Health** | **3.2**  **Engage in Effective Partnerships** |  |  |  |
|  | *3.2c*  *Use culturally and linguistically responsive communication strategies* | Apply culturally sensitive communication strategies | Interactive Class Activity: Review language translator programs available in local health systems and identify how to utilize these in everyday practice | Faculty lead discussion regarding pros and cons of each translator program and peer/faculty feedback on strategies for implementation |
|  | **3.5 Demonstrate Advocacy Strategies** |  |  |  |
|  | *3.5a*  *Articulate a need*  *for change* | Identify Healthy People 2030 Objectives that support improvement of health literacy.  Utilize various communication techniques to identify limited health literacy | Interactive Class Activity: Students identify Healthy People 2030 objectives that relate to health literacy (ODPHP, n.d.)  Simulation: Students alternate interviewing each other using different techniques (e.g. motivational interviewing) | Faculty led debriefing with interactive discussion using the PEARLS Healthcare Debriefing Tool (Bajaj et al., 2018) |
|  | *3.5b*  *Describe proposed change* | Identify strategies to improve health literacy | Interactive Class Activity: Students work in groups to edit health education materials to meet current recommendations for grade level and evaluate using the AHRQ *Health Literacy Universal Precautions Toolkit* (Brach, 2024), SMOG tool (Harvard T.H. Chan School of Public Health, 2025b), Flesch-Kincaid Grade Level (Kincaid et al., 1975), Fry Graph Formula Tool (Fry,1968) and PEMAT (Agency for Healthcare Research and Quality, 2020). | Student presentation to peers with peer and faculty feedback |
|  | *3.5e*  *Evaluate the effectiveness of advocacy* *actions* | Understand the importance of engaging clients in the use of health information technology  Assess current public health legislature regarding health literacy | Interactive Class Activity: Students work in groups to identify healthcare technology that improves health literacy and demonstrate how to utilize the technology  Interactive Class Activity: Students write letters to representatives supporting or not supporting current  health legislation | Faculty led debriefing with interactive discussion using the PEARLS Healthcare Debriefing Tool (Bajaj et al., 2018) |
| **Domain 4: Scholarship for the Nursing**  **Discipline** | **4.2**  **Integrate Best Evidence into**  **Nursing Practice** |  |  |  |
|  | *4.2c Use best evidence in practice* | Identify current best practices from peer reviewed journal articles and strategies for use in practice | Interactive Class Activity: Present 3-minute comparison of best practice recommendations and apply to case studies | Faculty led debriefing with interactive discussion using the PEARLS Healthcare Debriefing Tool (Bajaj et al., 2018) |
|  | *4.2d*  *Participate in implementation of a practice change to* *improve individual care* | Apply evidence-based practice to the community health setting | Clinical: Participate in a community health fair and provide education using evidence-based practice supported health literacy materials | Faculty led debriefing with interactive discussion using the PEARLS Healthcare Debriefing Tool (Bajaj et al., 2018) |
| **6.4: Interprofessional Partnerships** | *4.2e*  *Participate in evaluation of outcomes and their implications for practice*  ***6.4***  ***Work with other professions to maintain a climate of mutual learning, respect, and shared values***  *6.4d Collaborate with interprofessional (IPE) team members to establish mutual healthcare goals for individuals, communities, or populations.* | Analyze community activity results from community activity  Assess mutual healthcare goals to meet individual assessments | Clinical: Collect and review data from community health fair participants surveys including formative and summative evaluation  Clinical: Participate in interprofessional team home visits and perform an assessment to identify comprehensive individual needs.  Clinical: Collaborate with students from different disciplines to create an interprofessional plan of care for individuals in the home setting.  Interactive Class Activity: Collaborate with students from different disciplines and present case study from home visit. | Student presentation to peers with peer and faculty feedback  Student IPE collaboration with faculty feedback  Group presentations with peer and faculty feedback |
| **Domain 8: Informatics and Health Care Technologies** | ***8.2***  ***Use Information and Communication Technology to Gather Data, Create Information and Generate***  ***Knowledge*** |  |  |  |
|  | *8.2d*  *Demonstrate the appropriate use of health information literacy assessments and improvement strategies* | Apply knowledge of available health literacy tools to the community/public health setting | Clinical: Participate in a community health fair and provide education using evidence-based practice supported health literacy materials  Clinical: Students complete self-reflection on using health literacy education in the clinical setting | Faculty led debriefing with interactive discussion using the PEARLS Healthcare Debriefing Tool (Bajaj et al., 2018)  Discussion board with peer and faculty feedback on self-reflection |
|  | *8.3d*  *Examine how emerging technologies influence healthcare delivery and clinical decision-making* | Apply critical thinking skills to critique the accuracy of AI generated data. | Case Study: Provide research articles with case study scenarios. Students utilize AI-generated information to develop an individual plan of care. Compare the generated data to evidence-based recommendations and review for potential bias of marginalized populations.  Case Study: Provide research articles with case study scenarios. Students utilize Google to obtain information to develop an individual teaching plan. Compare the information retrieved from Google to evidence-based recommendation and review for accuracy. | Student presentation to peers with peer and faculty feedback.  Clinical Practice: Students complete self-reflection journal assignment highlighting the ethical dilemma created when using AI generated care plan for an individual.  Student presentation to peers with peer and faculty feedback.  Clinical Practice: Students complete self-reflection journal assignment highlighting the ethical dilemma created when using Google information for teaching plan for an individual. |

*Note. 1.*These educational strategies align with *The Essentials: Core Competencies for Professional Nursing Education* to provide examples for nursing instructors to incorporate into the classroom.

2. 6.4 Interprofessional education can be integrated into the other elements of this table.
